# Mobility of a Mononucleotide within a Lipid Matrix: A Neutron Scattering Study

**DOI:** 10.3390/life7010002

**Published:** 2017-01-04

**Authors:** Loreto Misuraca, Francesca Natali, Laura da Silva, Judith Peters, Bruno Demé, Jacques Ollivier, Tilo Seydel, Valerie Laux-Lesourd, Michael Haertlein, Giuseppe Zaccai, David Deamer, Marie Christine Maurel

**Affiliations:** 1 Institut Laue Langevin (ILL), 71, Avenue des Martyrs, 38000 Grenoble, France; misuraca.loreto@gmail.com (L.M.); jpeters@ill.fr (J.P.); deme@ill.fr (B.D.); ollivier@ill.fr (J.O.); seydel@ill.fr (T.S.); laux@ill.fr (V.L.-L.); haertlein@ill.fr (M.H.); zaccai@ill.fr (G.Z.); 2 Dipartimento di Fisica e Chimica, Università degli Studi di Palermo, Viale delle Scienze, Ed. 17, 90128 Palermo, Italy; 3 Consiglio Nazionale delle Ricerche, Istituto Officina dei Materiali (CNR-IOM), Research Unit in Grenoble, 71 Avenue des Martyrs, 38000 Grenoble, France; 4 Institut de Systematique, Évolution, Biodiversité, (ISYEB) UMR 7205 CNRS-MNHN-UPMC-EPHE Sorbonne Universités, CP50, 57 rue Cuvier, 75005 Paris, France; lddasilvalaura@gmail.com (L.d.S.); marie-christine.maurel@upmc.fr (M.C.M.); 5 Université Grenoble Alpes (UGA), UFR PhITEM, 621 Avenue Centrale, 38000 Grenoble, France; 6 Institut de Biologie Structurale (IBS), 71 Avenue des Martyrs, 38000 Grenoble, France; 7 Commissariat à l'énergie atomique et aux énergies alternatives (CEA), 17 Avenue des Martyrs, 38054 Grenoble, France; 8 Centre National de la Recherche Scientifique (CNRS), 25 Avenue des Martyrs, 38000 Grenoble, France; 9 University of California, Santa Cruz, CA 95060, USA; deamer@soe.ucsc.edu

**Keywords:** neutron scattering, multilamellar lipid matrix, mononucleotide mobility, hydration

## Abstract

An essential question in studies on the origins of life is how nucleic acids were first synthesized and then incorporated into compartments about 4 billion years ago. A recent discovery is that guided polymerization within organizing matrices could promote a non-enzymatic condensation reaction allowing the formation of RNA-like polymers, followed by encapsulation in lipid membranes. Here, we used neutron scattering and deuterium labelling to investigate 5′-adenosine monophosphate (AMP) molecules captured in a multilamellar phospholipid matrix. The aim of the research was to determine and compare how mononucleotides are captured and differently organized within matrices and multilamellar phospholipid structures and to explore the role of water in organizing the system to determine at which level the system becomes sufficiently anhydrous to lock the AMP molecules into an organized structure and initiate ester bond synthesis. Elastic incoherent neutron scattering experiments were thus employed to investigate the changes of the dynamic properties of AMP induced by embedding the molecules within the lipid matrix. The influence of AMP addition to the lipid membrane organization was determined through diffraction measurement, which also helped us to define the best working Q range for dynamical data analysis with respect to specific hydration. The use of different complementary instruments allowed coverage of a wide time-scale domain, from ns to ps, of atomic mean square fluctuations, providing evidence of a well-defined dependence of the AMP dynamics on the hydration level.

## 1. Introduction

Several scenarios related to the first steps of the origins of life have for the most part been investigated independently from one another. Examples include the lipids that are essential for the early stages of compartmentation, and RNA which has been proposed to have preceded life based on DNA–RNA and proteins [1]. Today, the borders are becoming blurred and we have realized not only that RNAs can function within lipid compartments, but also that molecular interactions between lipids and RNAs help to promote the synthesis of RNAs. RNA has the dual capacity of being a carrier of genetic information and a catalyst of chemical transformations, and is therefore a convincing candidate as an ancestral biopolymer [2,3,4,5]. However, it is not clear how a non-biological process could have synthesized random polymers of RNA-like molecules as a first step toward living systems.

Non-enzymatic syntheses of RNA-like polymers from ordinary mononucleotides have been recently studied in simulated prebiotic conditions undergoing cycles of hydration–dehydration at an elevated temperature [6,7,8]. Those conditions simulated hydrothermal processes that commonly occur in volcanic hydrothermal fields today and were presumably ubiquitous on the early Earth. In addition, the inclusion of a multilamellar phospholipid as an organizing matrix allows the synthesis of short polymeric products, presumably because the liquid crystalline matrix serves to concentrate and organize the mononucleotides and also allows a certain degree of diffusional mobility required for extensive polymerization. The chemical potential driving polymerization is provided by an anhydrous phase in which water becomes a leaving group that allows phosphodiester bonds to link the monomers through a condensation reaction. The idea of guided polymerization dates back more than forty years [9,10,11], but there were no studies of the arrangement of nucleotides within an organized matrix such as the 1,2-dimyristoyl-sn-glycero-3-phosphocholine (DMPC) investigated by Toppozini et al. [12].

Our primary objective in the present study was to determine how mononucleotides are organized within a multilamellar lipid structure that is produced when liposomes and solutes undergo controlled dehydration. X-ray diffraction results [12] confirmed that the AMP molecules are highly entangled, with the phosphate and ribose groups in close proximity to each other, a structure that may facilitate polymerization of the nucleotides into RNA-like polymers. We extended and compared polymerization of mononucleotides within a multilamellar lipid matrix using neutron scattering so as to investigate 5′-adenosine monophosphate (AMP) molecules captured in a multilamellar phospholipid matrix composed of a mixture of phospholipids extracted from *Pichia pastoris* cells [13]; this could help to understand the formation of RNA-like polymers.

The aim of the work reported here was to determine and compare how mononucleotides are captured and differently organized within matrices and multilamellar phospholipid structures and to explore the role of water in organizing the system to determine at which level the system becomes sufficiently anhydrous to lock the AMP molecules into an organized structure and initiate ester bond synthesis. We used neutron scattering to investigate AMP molecules captured in a multilamellar phospholipid matrix composed of a mixture of phospholipids extracted from *Pichia pastoris* cells [13] to study the formation of RNA-like polymers. Elastic incoherent neutron scattering experiments (EINS) were employed to investigate the changes of dynamical properties of AMP induced by embedding the molecules within the lipid matrix. The use of different complementary instruments allowed covering the time-scale domain, from ns to ps, of atomic mean square fluctuations, providing evidence of the well-defined dependence of the AMP dynamics on the hydration level. Neutrons are scattered mainly by H nuclei, which, on this time scale, reflect the motion of the chemical groups to which they are bound.

## 2. Materials and Methods

### 2.1. Sample Preparation

#### 2.1.1. Lipid Extraction

The cross section of ^1^H strongly dominates incoherent neutron scattering and changes in scattering amplitudes are achieved through the substitution of hydrogen by its isotope, deuterium. This allows contrast variation methods [14] to either highlight or match out the scattering from particular regions of a system. Deuterated lipids and D_2_O were used with natural abundance AMP in order to focus on the motions of the AMP molecules in the inter-bilayer space at high and low hydration levels. Perdeuterated lipids were extracted from methylotrophic yeast *Pichia pastoris* grown at 30 °C. Fully deuterated minimal medium based on basal salt medium, pH 6.0 (*Pichia* fermentation process guidelines, Invitrogen, Waltham, MA, USA) containing 20 g·L^−1^ d_8_-glycerol (Euriso-Top, Saint-Aubin, France) as carbon source was used. *P. pastoris* cells were harvested by centrifugation at the end of the exponential phase at an optical density OD_600_ of about 80. For comparison purposes, hydrogenous extracts obtained under the same conditions but using hydrogenous glycerol and H_2_O in the growth media, have also been analyzed. Compositional analyses of both types of extract have been described by de Ghellinck et al., [13]. Deuterated yeast cells were produced in the Deuteration laboratory with ILL’s Life Sciences Group, harvested and poured in boiled ethanol in order to block the action of endogenic enzymes able to hydrolyze glycerophospholipids. Lipids were then extracted according to the Folch method [15]. The system was hydrated under isopiestic conditions at 100% relative humidity in a desiccator with 99.9% D_2_O.

#### 2.1.2. Preparation of Matrix/AMP Complexes

Small lipid vesicles (liposomes) were prepared by dispersing the previously extracted lipids in ultra-pure water to produce concentration of 10 mM. The milky solution (deuterated multilamellar phospholipid liposomes, dMPL), which initially contained multilamellar vesicles, was sonicated for 15 min until the solution became transparent, indicating that small unilamellar vesicles are formed. The 5′-adenosine monophosphate (AMP) monohydrate powder was added to ultra-pure water in 10 mM and 20 mM concentrations and heated in a bath until completely dissolved. The AMP solution and the dMPL suspension were then mixed in molar ratios of 1:2.

Mixtures of 300 mg of dMPL and 150 mg natural abundance AMP were heated to dryness. The desired hydration levels *h* = 0%, *h* = 15%, *h* = 25% and *h* = 35% (where *h* = 100 × [gr H_2_O]/[gr sample]) were achieved monitoring the mass change of the sample exposed to D_2_O saturated atmosphere. Finally, they were placed in vacuum tight aluminum sample holders of 0.2 mm neutron path length.

Samples will be noted hereafter as AMP-dMPL-h0, AMP-dMPL-h15, AMP-dMPL-h25 and AMP-dMPL-h35. Knowing the molecular weights of AMP (≈347 g/mol) and MPL (≈427 g/mol), weighted with their percentage composition [13] the calculated relative contributions to the total cross-section (with dMPL incoherent cross section less than 10% of the total) allow us to neglect the matrix contribution and to ascribe any eventual dynamical changes univocally to the AMP molecules.

### 2.2. Experiments

Three different neutron scattering instruments were used for this work [16,17]:

- The cold Neutron Backscattering Spectrometer IN16b is a high energy resolution instrument, so it is particularly devoted to discriminating slow molecular motions; in particular, IN16b provides an energy resolution of ∆*E ≈* 0.8 µeV Full Width at Half Maximum (FWHM), corresponding to an observable time scale of ≈1 ns [18]. The scattering vector Q, given by the vectorial sum of the incident and scattered neutron wave vectors and corresponding to the analogous in the reciprocal Fourier space of the distance in the direct space, is measured on IN16b in the range (0.3 < Q < 1.8 Å^−1^), while the incident wavelength has been fixed to *λ* = 6.27 Å; elastic scans were performed stepwise in the temperature range (20 < T < 310 K).
- The thermal Neutron Backscattering Spectrometer IN13 is similar to the former, but with different energy resolution and observable Q range, ∆*E ≈* 8 µeV FWHM leading to *t ≈* 0.1 ns and (0.5 < Q < 4.9 Å^−1^). By using the CaF_2_ monochromator, the incident wavelength has been fixed to the value *λ* = 2.23 Å; elastic scans were performed stepwise in the temperature range (20 < *T* < 310 K).
- The disk Chopper Time-of-flight Spectrometer IN5 is a direct geometry time-of-flight instrument primarily devoted to inelastic/quasielastic neutron scattering measurements (INS/QENS), thus allowing to measure low-energy transfer processes as a function of the momentum transfer. For this experiment, QENS measurements have been performed for all the samples with two incident wavelengths: (a) *λ* = 5 Å, with an accessible Q interval of (0.4 < Q < 2.2 Å^−1^), providing an energy resolution of ∆*E* ≈ 100 µeV FWHM and an observable time-scale of ≈5 ps; (b) *λ* = 10 Å, with (0.1 < Q < 1 Å^−1^), ∆*E* ≈ 10 µeV FWHM and an observable time-scale of ≈50 ps. Elastic scans were determined stepwise in the temperature range (20 < T < 310 K); quasi-elastic spectra were taken at T = 310 K. Furthermore, IN5 also allows us to access diffraction patterns for qualitative investigations. Thus, diffraction scans were acquired at variable relative humidity and temperature. Moreover, the empty cell contribution has been removed.

In order to justify the assumption that multiple scattering events can be neglected, the transmission of all the samples used has been verified to be ≈95%. All ENS/QENS measurements were also corrected for energy-dependent detector efficiency, empty cell scattering and sample absorption. Normalization was performed with respect to low T measurements (T = 20 K) in the case of elastic scans, or with respect to Vanadium spectra (a totally inelastic scatterer) in the case of quasi-elastic data.

### 2.3. Elastic and Quasi-Elastic Incoherent Neutron Scattering Data Analysis

The output arising from an elastic scan is the low T normalized elastic scattering intensity measured at zero energy exchange, denoted as I(Q, T)/I(Q, T = 20 K). A first, model-independent, qualitative analysis can be obtained by integrating the intensities over Q to see how the obtained information on the fraction of the scatterers in the sample, which is immobile on the observation time scale, behaves as a function of the temperature. Also, the Q-dependence of the elastic scans can be used to calculate mean square displacements (MSD) <*u*^2^>: this can be defined, for our purposes, as the result of all contributions to the atomic motions (vibrational, rotational, translational motions) acting on timescales commensurate with and beyond the instrumental resolution. To calculate it from the datasets, the elastic scattering intensity I(Q, T)/I(Q, T = 20 K) was analyzed in the framework of the Gaussian approximation, according to the following definition [19]:

(1) $$\frac{I\left(Q,\;T\right)}{I\left(Q,\;T=20\;K\right)}≅e^{-\frac{\langle u^{2}\rangle }{6}Q^{2}}$$

In practice, <*u*^2^> is determined by fitting the Q^2^ dependence of *ln*[I(Q, T)/I(Q, T = 20 K)] with a straight line over a suitable Q^2^ range, verifying for each case that the condition <*u*^2^> Q^2^ ≤ 2 was obeyed [20].

Regarding the quasi-elastic data analysis, the output obtained after corrections is a number of S(∆*E*) spectra, each one corresponding to a defined Q range (spectra are grouped in Q to improve the statistics and to allow reliable curve fits). A simple and model-independent comparison between the different hydration samples can be provided by integrating S(∆*E*) spectra all over Qs (far enough from the Bragg peaks) and comparing the obtained normalized spectra. For the sake of manuscript length, a detailed analysis of the QENS data is not provided here, and it will be the object of a dedicated paper currently in preparation.

## 3. Results

A neutron scattering spectrum contains two distinct contributions: (1) diffraction peaks from the lamellar organization of the sample and (2) a so-called incoherent contribution on which dynamical analysis is performed. It was important, therefore, before performing the dynamical analysis, to remove the diffraction peaks from the spectra. Moreover, on top of lamellar diffraction rings, in the highly hydrated sample at low temperature, the typical diffraction peaks of crystalline ice (1.6 < Q < 1.8 Å^−1^) were also observed, confirming the presence of free water (D_2_O).

**Figure 1:**
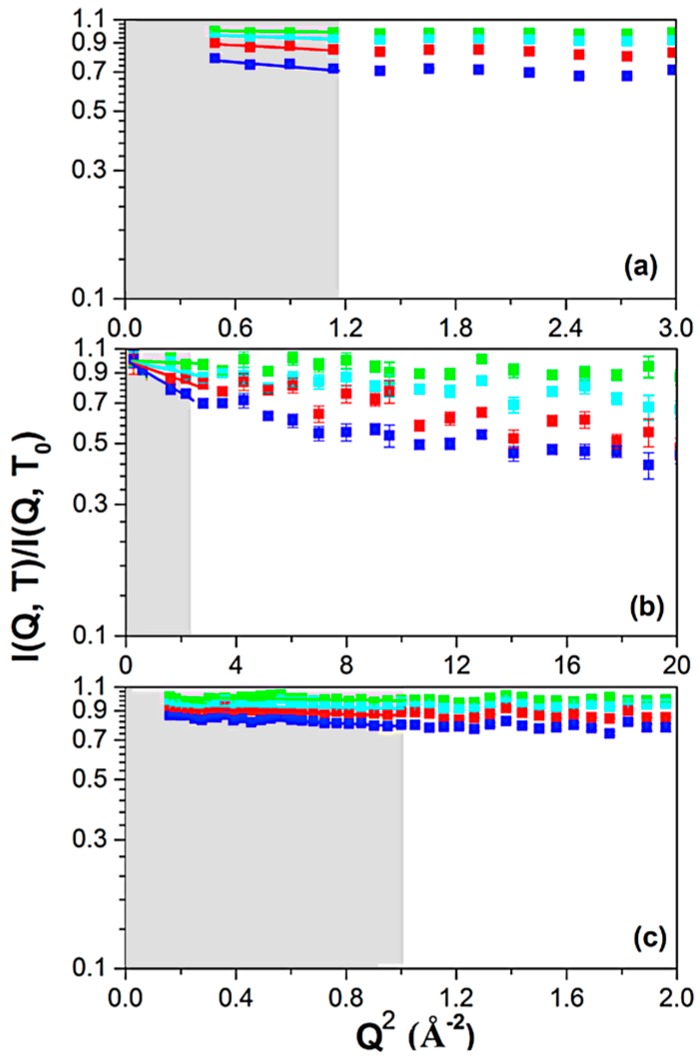
Q^2^ dependence of the normalized elastic scattering intensity curves for the AMP-dMPL-h0 sample, obtained with the neutron spectrometers (**a**) IN16b, with observed time-scale *t ≈* 1 ns; (**b**) IN13, with observed time-scale *t ≈* 0.1 ns; (**c**) IN5, with observed time-scale ≈ 5 ps. Curves are normalized to T = 20 K for IN16b and IN13, T = 50 K for IN5. For the sake of clarity, only a few selected temperatures as shown: 80 K (**green**), 160 K (**cyan**), 240K (**red**) and 300 K (**blue**). The ranges used in further analysis are indicated in **grey**.

The use of the 3 instruments allowed us to explore a time-length domain from 1 ns (IN16b) to 5 ps (IN5) and from 1 to about 10 Å. The procedure allowed us to choose the proper Q range to be investigated, and the following samples were measured on IN16b, IN13 and IN5: AMP-dMPL-h0, AMP-dMPL-h15, AMP-dMPL-h25 and AMP-dMPL-h35. Examples of the data plotted as the Q^2^ dependence of the normalized elastic intensities (I) from which MSD will be derived (see Methods) are shown for the three instruments in Figure 1. For clarity, only a few selected temperatures are shown (80, 160, 240 and 300 K). The three instruments clearly address different Q^2^ ranges as presented on the *x*-axes: Q^2^ < 3.0, 20 and 4.0 Å^−2^ for IN16b, IN13 and IN5, respectively. In contrary, the chosen *y*-axis is identical for all three instruments and shows that the measured intensities are the most broadly distributed on IN13, where we have the highest accessible Q-range, and still more spread on IN16b than on IN5 due to the larger time window. The normalized intensities summed over all Q vs. temperature for the 4 different hydration levels with data taken from IN16b, IN13, IN5 respectively are reported in the Supplementary Information (SI).

**Figure 2:**
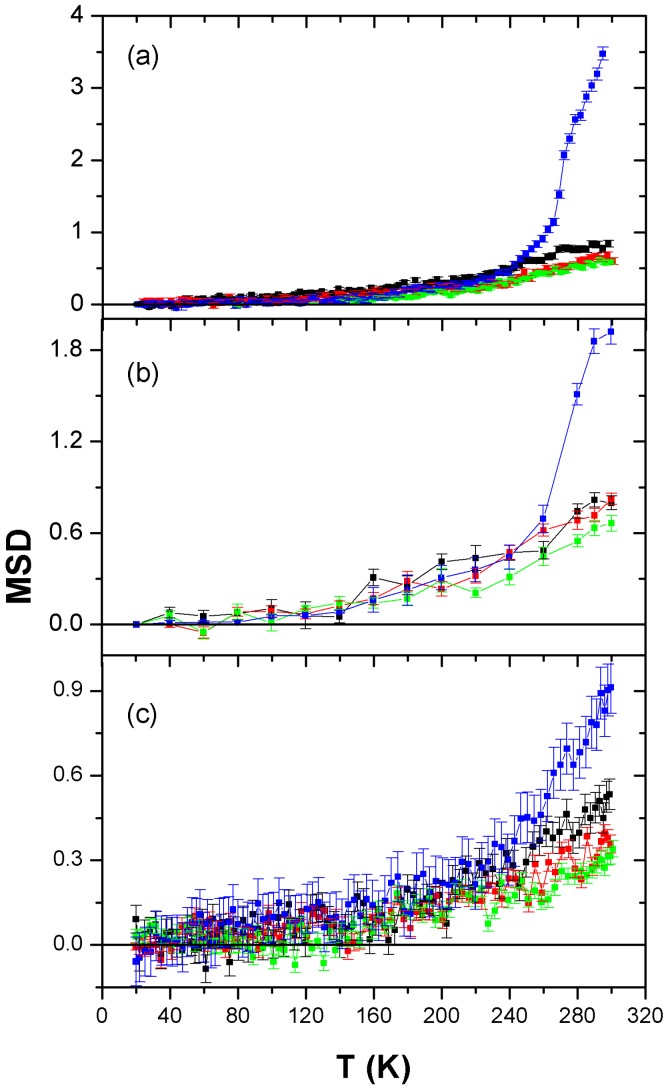
MSDs of AMP-dMPL at different hydrations (h0 in **green**, h15 in **red**, h25 in black and h35 in **blue**) extracted from IN16b (panel (**a**)); IN13 (panel (**b**)); and IN5 (panel (**c**)) data.

To look at a small Q interval means to detect large-scale movements; the corresponding MSD graphs are reported in Figure 2, in which values have been extracted from the I vs. Q fit, using Equation (1) in the Q region shown in grey in Figure 1.

In a simplistic picture, we consider the atoms to be held together by springs. At low temperature these springs are harmonic, yielding MSDs that increase linearly with temperature. At a so-called dynamical transition temperature, the springs soften and the atoms acquire greater mobility [21]. The occurrence of such a dynamic transition is observed at about 150 K for all timescales explored and all sample hydrations.

From Figure 2, one can easily argue that the dynamics of the AMP-dMPL-h35 sample differ significantly from that of the other sample hydrations, suggesting the occurrence of additional dynamical transitions. We recall that the signal is largely dominated by the motions of the AMP molecules in the sample, because both lipids and water are deuterated. Thus, Figure 2 clearly shows an enhancement of the AMP mobility as hydration is increased in the sample.

**Figure 3:**
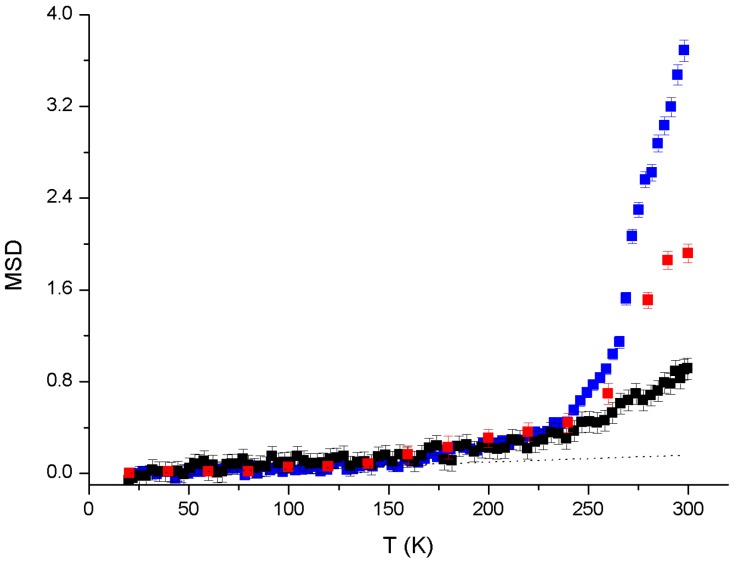
MSDs of the AMP-dMPL-h35 sample as calculated from IN16b (**blue**), IN13 (**red**) and IN5 (**black**) data. The dotted line is the harmonic contribution.

To better determine at which temperature these further transitions take place, the MSDs of the AMP-dMPL-h35 sample for the three instruments used are shown in Figure 3, with the approximately linear harmonic contribution also reported for comparison.

In agreement with the literature [19,22,23,24], the first hydration-independent transition, observed above 150 K, corresponds to activation of CH_2_ groups, which may induce further mobility to the AMP. A second transition is then observed at about 240 K associated with an enhancement of AMP mobility for the three instruments, indicating the crossing of the same activation barrier in the energy landscape with larger amplitude motions becoming apparent at the longer timescales and for the higher hydration values. Further inspection suggests that the IN16b and IN13 data (less evident on IN5) show an additional transition at T = 270–280 K, rationally assigned to acquired proton mobility induced by the melting of free water.

The enhancement of AMP dynamics at the h35 hydration level is further confirmed by a visual inspection of QENS data acquired for all samples on IN5 and here reported exclusively for qualitative investigation. Detailed data analysis is currently in progress and the results will be the subject of a future dedicated paper.

**Figure 4:**
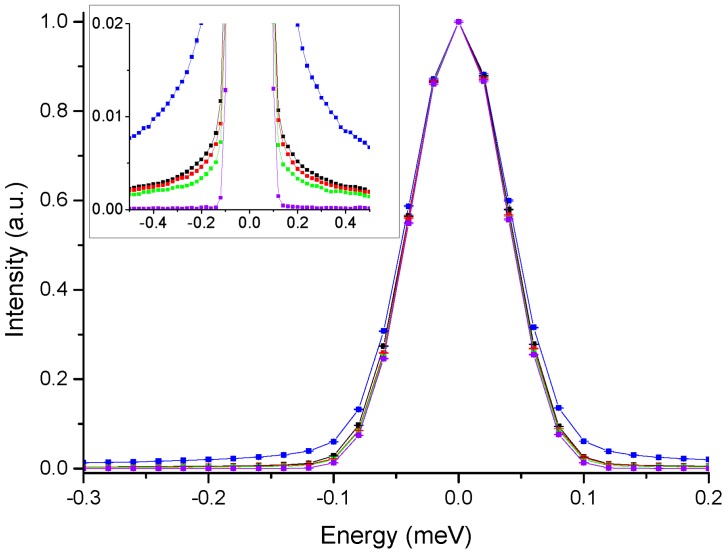
Normalized QENS spectra integrated all over Q values for AMP-dMPL at different hydrations (h0 in **green**, h15 in **red**, h25 in **black** and h35 in **blue**) acquired on IN5. Vanadium spectrum is also plotted for comparison (violet).

Figure 4 shows, for the four hydrations, the normalized QENS spectra summed over all Qs.

**Figure 5:**
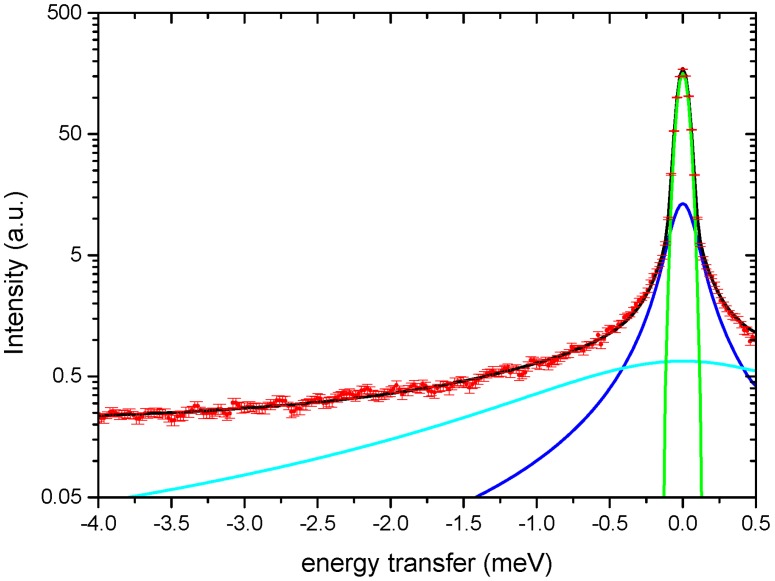
QENS spectrum AMP-dMPL-h35 acquired at Q = 1.00(3) Å^−1^ fitted by the sum of a Dirac’s delta (**green**) and two Lorentzians (**light blue** and **blue**), where each curve is already convoluted with the instrumental resolution), plus a flat background (not shown). **Red** symbols indicate the experimental points and the corresponding error bars; black curve is the total fitting curve.

The much broader QENS contribution observed for the AMP-dMPL-h35 sample is a clear indication of enhanced mobility. To confirm the presence of free water molecules, we report in Figure 5 an example of a QENS spectrum for AMP-dMPL-h35 acquired at Q = 1.00(3) Å^−1^, fitted by the instrumental resolution (green) and 2 Lorentzian functions (minimum number of curves to correctly reproduce the experimental data).

The broader Lorentzian half width at half maximum (HWHM) agrees well with typical values for free water [25]. The additional Lorentzian may provide information on the motion of H atoms from AMP molecules which can be directly related to what is observed with the elastic scans.

## 4. Discussion

Previous studies have reported that guided polymerization within organizing matrices can promote non-enzymatic condensation reactions leading to the synthesis of RNA-like polymers. Here, we used neutron scattering and deuterium labelling to investigate the mobility of 5′-adenosine monophosphate (AMP) molecules captured in a multilamellar phospholipid matrix. The role of water in organizing matrices was explored to determine at which hydration level the system modifies the diffusion of AMP molecules and locks them into an organized structure to initiate ester bond synthesis.

The neutron scattering spectrum contains two distinct contributions: (1) diffraction peaks from the lamellar organization of the sample; (2) a so-called incoherent contribution on which dynamical analysis is performed. The diffraction peaks were removed from the spectra before analyzing the dynamic properties of AMP within the matrix. AMP-lipid samples at three different hydrations (*h* = 0%, 15%, 25%, 35%) were examined at three timescales (5 ps, 0.1 ns, 1 ns). In all samples the lipids and water were deuterated so that the neutron scattering signal was dominated by the motions of AMP molecules. MSDs were measured for a broad temperature range (20 K to 300 K), and the main main dynamical transition (significant softening) was observed for the three timescales close to 240 K, indicating the crossing of the same activation barrier in the energy landscape with larger amplitude motions becoming apparent at the longer timescales and for the higher hydration values, with a significant jump in MSD between the hydrations 25% and 35%. Ice diffraction peaks observed in the 35% hydrated sample indicated the presence of free water, which was confirmed by an initial analysis of the QENS spectrum of the sample. The ice melting was also observed to trigger a transition in AMP MSD at about 275 K for the h35 sample.

We can now relate the neutron scattering results to the conditions leading to polymerization of mononucleotides. The experiments described here were performed over a temperature regime of 20 K to 300 K during exposure of the lipid-AMP matrix to h from 0% to 35%. The MSD results shown in Figure 2 and Figure 3 clearly indicate that AMP becomes mobile and enters a diffusion regime at T > 275 K and *h* > 35%. The polymerization studies were performed at 358 K with cycles from 0% relative humidity (RH) (dry carbon dioxide) to 100% RH (addition of bulk water), conditions designed to simulate natural hydration-dehydration cycles characteristic of hydrothermal fields. The question then concerns the physical state of the AMP in the dehydration phase when dry carbon dioxide at 0% RH is passed over the samples at 358 K. It is obvious that no free water will remain in the sample under those conditions. Furthermore, from previous studies using X-ray diffraction [12,26], it is also clear that the AMP in the lipid matrix is not in a disordered glassy state but instead has a significant degree of order, either as paracrystalline arrays (pure AMP) or with base stacking (AMP:UMP in a 1:1 mole ratio mixture). This degree of order could not be present if the nucleotides were freely diffusing, in agreement with the neutron scattering results at *h* < 35%.

In summary, the neutron scattering results provide additional insight into the behavior of nucleotides such as AMP during cycles of hydration and dehydration at elevated temperatures. Dehydration allows the self-assembly of the lipids into a multilamellar matrix that captures the AMP between bilayers of what is essentially a liquid crystalline multilamellar matrix, and intermolecular forces allow the AMP to assemble into orderly arrays. Over time, bulk water evaporates to produce a relatively anhydrous matrix in which AMP is still able to diffuse slowly, and finally condensation reactions and polymerization are initiated by synthesis of ester bonds. The free energy required for condensation reactions is made available by the chemical potential resulting from molecular crowding [27] and by the reduction of entropy as AMP is forced into orderly arrays. Upon rehydration, when the water content reaches 35% the AMP is able to diffuse much more freely and, further increasing the hydration, will be captured in vesicles together with any polymers that have been synthesized.

## Supplementary Materials

The following are available online at http://www.mdpi.com/2075-1729/7/1/2/s1. Figure S1: Normalized elastic scattering intensities.

## Abbreviations

The following abbreviations are used in this manuscript:

AMP	5′-adenosine monophosphate
dMPL	deuterated multilamellar phospholipid liposomes
DNA	Deoxyribonucleic acid
EINS	Elastic incoherent neutron scattering
FWHM	Full Width at Half Maximum
HWHM	Half Width at Half Maximum
MDPI	Multidisciplinary Digital Publishing Institute
MSD	Mean Square Displacement
*Q*, *Q*^2^	Moment transfer and its square
QENS	Quasi-elastic neutron scattering
RH	Relative humidity
RNA	Ribonucleic acid
