# Supplementary material for: Mobility of a Mononucleotide within a Lipid Matrix: A Neutron Scattering Study
Source: Life (Basel). 2017 Jan 4;7(1):2. doi: 10.3390/life7010002 (PMC5370402; doi:10.3390/life7010002)
Supplement: Supplementary file 1 [file life-07-00002-s001.pdf]

Supplementary Materials Mobility of a Mononucleotide within a Lipid Matrix: A Neutron Scattering Study

Loreto Misuraca, Francesca Natali, Laura da Silva, Judith Peters, Bruno Deme, Jacques Ollivier, Tilo Seydel, Valerie Laux, Michael Haertlein, Giuseppe Zaccai, David Deamer and Marie Christine Maurel

1. Elastic Scattering Intensities

Figure S1 reports the temperature dependence of the normalized elastic intensities integrated all over Q values for AMP-dMPL at the four hydrations acquired on IN16b, IN13 and IN5, respectively (in descending of energy resolution). Summed all over Qs is intended until a value of Q ≈ 1 $Å^{-1}$: this is due to the experimental observation of a multiplicity of Bragg peaks, as already shown in Figure S1; the only exception is I(T)/I(20 K) intensity scan for IN13 to improve the statistics (IN13 has only 3 detectors with a Q value below 1 $Å^{-1}$).

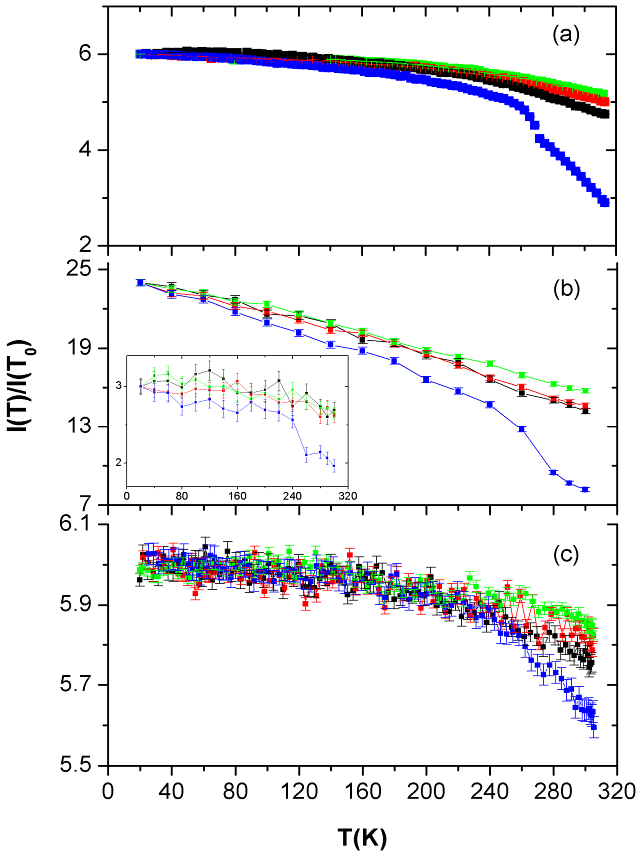

Figure S1. Normalized elastic scattering intensity curves integrated all over Q values for AMP-dMPL-h0 (**green**), AMP-dMPL-h15 (**red**), AMP-dMPL-h25 (**black**) and AMP-dMPL-h35 (**blue**), obtained with the neutron spectrometers (**a**) IN16b; (**b**) IN13 and (**c**) IN5. Curves are normalized to T = 20 K for IN16b and IN13, T ≈ 50 K for IN5. Inset in panel b: integration over first 3 detectors for check.
